# Administration of a herbal formulation enhanced blastocyst implantation via IκB activation in mouse endometrium

**DOI:** 10.1186/s13020-020-00395-x

**Published:** 2020-10-20

**Authors:** Songhee Jeon, Quan Feng Liu, Hua Cai, Ha Jin Jeong, Su-Hyun Kim, Dong-Il Kim, Ju-Hee Lee

**Affiliations:** 1 Department of Biomedical Sciences, Center for Creative Biomedical Scientists at Chonnam National University, Gwangju, 61469 Republic of Korea; 2 Department of Neuropsychiatry, Graduate School of Korean Medicine, Dongguk University, Gyeongju, 38066 Republic of Korea; 3 Department of Obstetrics & Gynecology, College of Korean Medicine, Sangji University, Wonju, Gangwon-do 26338 Republic of Korea; 4 Department of Obstetrics & Gynecology, College of Korean Medicine, Dongguk University Ilsan Hospital of Korean Medicine, Goyang, Gyeonggi-do 10326 Republic of Korea; 5 College of Korean Medicine, Dongguk University, Goyang, Gyeonggi-do 10326 Republic of Korea

**Keywords:** Herbal formulation, RU486, Matrix metalloproteinases, Blastocyst implantation, IκB

## Abstract

**Background:**

BaelanChagsangBang (BCB), a herbal formulation consisting of eleven herbs, may be prescribed as a reproductive functional supplement to improve ovulation and implantation during the treatment of infertility and recurrent abortion in Korean Medicine. This study aimed to investigate the effects and action mechanisms of water-extracted BCB on endometrial receptivity and blastocyst implantation under normal conditions and in a mifepristone (RU486)-induced implantation failure murine model.

**Methods:**

In vitro, the antioxidant potentials of BCB were evaluated using DPPH and superoxide anion radical scavenging assays and a DCFH-DA assay, and the cytotoxic and cytoprotective effects of BCB were confirmed using an MTT assay. In vivo, C57BL/6 female mice (n = 6 per group) orally received BCB (300 mg/kg/day), a dose similar to that used clinically, from 7 days before pregnancy until the end of the experiment. On day 4 of pregnancy, RU486 (4 mg/kg) was injected subcutaneously to induce implantation failure. The effect of BCB on embryo implantation was evaluated by implantation rate analysis, histological examination, and western blotting of uterus tissues.

**Results:**

BCB water extract showed strong anti-oxidative and cytoprotective effects in vitro. In vivo administration of BCB water extract increased the number of newborn pups in BCB-treated mice versus sham-treated mice under normal conditions and improved the number of implantation sites in pregnant mice despite RU486 injection. BCB increased the protein levels of cyclooxygenase-2 and inducible nitric oxide synthase through IκB activation. Moreover, the expression levels of matrix metalloproteinases at uterus implantation sites were up-regulated in the BCB-treated group as compared with those in the RU486-treated group.

**Conclusion:**

These results show BCB improved embryo implantation through IκB activation in our mouse model and suggest that BCB has therapeutic potential in the context of poor endometrial receptivity.

## Background

Implantation is a process by which the embryo (blastocyst stage) attaches to endometrium in early pregnancy and is only allowed during a short period called the ‘implantation window’. This process is regulated by complex and precise interactions between the embryo and endometrium [1], which involve differentiation to form a receptive endometrium under the influences of various biological factors of embryonic and maternal origin that include cytokines, growth factors, and adhesion molecules [2]. Implantation failure is considered to be the result of embryo defects, decreased endometrial receptivity, and embryo-uterine dialogue failure [3]. Infertility is an increasing problem among couples of reproductive age, and over the past three decades, an increasing number of married women have sought pregnancy assistance due to infertility [4–6]. Although assisted reproduction techniques (ART) have been widely used for the clinical treatment of infertility resulting from benign gynecological disorders or primary unexplained infertility, subsequent pregnancy rates remain unsatisfactory [4, 7]. In traditional Eastern medicine, herbal medicines, acupuncture, and moxibustion are used to treat female infertility [8, 9], and recently, due to accumulated scientific evidence, herbal medicine has emerged as an effective, complementary, alternative medicine for improving endometrial receptivity [10, 11].

In Eastern medicine, kidney deficiency, Qi and blood deficiencies, and liver congestion are considered to be the causes of infertility [12]. Thus, the most important treatments for infertility involve tonifying kidneys, reinforcing Qi, and nourishing blood to induce ovulation, improve oocyte quality, and generate a suitable endometrial environment [13]. BaelanChagsangBang (BCB) has been prescribed based on Wontogotae-tang in Bangyakhappyeon (a classical textbook of traditional Korean medicine) and Sutaehwan (Shou Tai Wan or Shoutai Pill) in *Yixue Zhongzhong Canxilu* (Records of Tradition Chinese and Western Medicine in Combination), as a supplement for reproductive system functions that can be used to improve ovulation and implantation during treatment for infertility and recurrent abortion [6, 14, 15]. BCB consists of a mixture of eleven herbs, that is, the seeds of *Cuscuta chinensis* Lam. (Dodder seed), the rhizomes of *Dioscorea japonica* Thunb., the fruits of *Rubus coreanus* Miq., the roots of *Panax ginseng* C. A. Mey., the fruits of *Lycium chinense* Mill., the roots of *Angelica gigas* Nakai, the leaves of *Perilla frutescens* L. Britton, the fruits of *Amomum villosum* Lour., the leaves of *Artemisia princeps* Pamp., the rhizomes of *Zingiber officinale* Roscoe, and the fruits of *Zizyphus jujuba* Mill. var. *inermis* Rehder. These BCB constituents are the main herbs used to tonify kidneys, reinforce Qi, and nourish blood. Although this prescription is used clinically to treat female infertility, experimental evidence obtained from in vitro and in vivo laboratory studies is lacking [6]. Mifepristone (also known as RU486) is a potent antiprogesterone agent, which can be used orally to induce abortion [16] or as an effective emergency contraceptive [17], and has often been used to induce embryo implantation failure or polycystic ovary syndrome in experimental animal models [10, 18, 19]. In the present study, we investigated the effects of a BCB water extract on endometrial receptivity and embryo implantation under normal conditions and in an RU486-induced mouse model of implantation failure.

## Methods

### Preparation of BCB

The constituents of BCB are shown in Table 1. All required herbs or herbal parts were purchased from Humanherb (Daegu, Korea), a good manufacturing practice (GMP)-certified Korean herbal medicine supplier. Voucher specimens are stored at the College of Korean Medicine, Dongguk University. To prepare the BCB extract, a mixture of the dried requisite parts of *Cuscutae Semen*, *Dioscoreae Rhizoma*, *Rubi Fructus*, *Ginseng Radix*, *Lycii Fructus*, *Angelicae Gigantis Radix*, *Perillae Folium*, *Amomi Fructus*, *Artemisiae Argyi Folium*, *Zingiberis Rhizoma Crudus*, and *Zizyphi Fructus* (weight ratio 8:16:10:4:4:2:4:4:4:3:2, respectively) was pulverized and extracted twice with 10 volumes of water at 85–90 °C for 3 h. The extract was then passed through a 50 μm filter paper, and the filtrate was concentrated by vacuum evaporation at 60 °C, lyophilized to yield BCB extract (yield 6.5% based on herbal mixture weight).

**Table 1:** Composition of BaelanChagsangBang

Latin name	Scientific name	Used part	Voucher specimen	Ratio
Cuscutae Semen	*Cuscuta chinensis* Lam. (Convolvulaceae)	Seed	DUMCKM2015-100	8
Dioscoreae Rhizoma	*Dioscorea batatas* Decne *Dioscorea japonica* Thunb. (Dioscoreaceae)	Rhizome	DUMCKM2015-036	16
Rubi Fructus	*Rubus coreanus* Miq. (Rosaceae)	Fruit	DUMCKM2015-110	10
Ginseng Radix	*Panax ginseng* C.A.Mey. (Araliaceae)	Root	DUMCKM2015-054	4
Lycii Fructus	*Lycium chinense* Mill./*Lycium barbarum* L. (Solanaceae)	Fruit	DUMCKM2015-008	4
Angelicae Gigantis Radix	*Angelica gigas* Nakai (Umbelliferae)	Root	DUMCKM2015-015	2
Perillae Folium	*Perilla frutescens* L. Britton (Labiatae)	Leaf	DUMCKM2015-055	4
Amomi Fructus	*Amomum villosum* Lour. (Zingiberaceae)	Fruit	DUMCKM2015-034	4
Artemisiae Argyi Folium	*Artemisia argyi* Lev. et Vant *Artemisia princeps* Pamp *Artemisia montana* (Nakai) Pamp. (Compositae)	Leaf	DUMCKM2015-101	4
Zingiberis Rhizoma Crudus	*Zingiber officinale* Roscoe (Zingiberaceae)	Rhizome	DUMCKM2015-004	3
Zizyphi Fructus	*Zizyphus jujuba* Mill. var. inermis Rehder (Rhamnaceae)	Fruit	DUMCKM2015-016	2

### BCB analysis by HPLC

Marker compounds in BCB extract were analyzed using a Dionex Ultimate 3000 HPLC system (Thermo Fisher Scientific, Waltham, MA, USA), equipped with a dual pump, an autosampler, a temperature regulated column oven, a diode-array spectrophotometric detector, and Chromeleon 6.8 chromatography management system software [20]. Ellagic acid (Sigma-Aldrich, St. Louis, Mo, USA) and chlorogenic acid (Sigma-Aldrich) were used as standards. Component separations were achieved on a VDSpher C-18 column (VDSoptilab, Germany) maintained at 30 °C. The column was step-gradient eluted using 0.3% trifluoroacetic acid (A) and acetonitrile (B) at a flow rate of 0.8 mL/min as follows: 10% B for 0–25 min, 60% B for 25–30 min, 100% B for 30–36 min, and 10% B for 36–40 min. Ellagic acid and chlorogenic acid were detected at 254 nm and 340 nm, respectively.

### Cell culture and cell viability

Chinese hamster ovary (CHO)-K1 cells were obtained from the Korean Cell Line Bank (Seoul, Republic of Korea) and cultured in DMEM (Dulbecco’s modified Eagle’s medium; WELGENE, Gyeongsan, Republic of Korea), supplemented with 10% fetal bovine serum (WELGENE) and 100 U/mL penicillin/100 μg/mL streptomycin (Thermo Fisher Scientific, Grand Island, NY, USA) at 37 °C in a humidified incubator (5% CO_2_/95% air; Thermo Fisher Scientific, Langenselbold, Germany). Cell viabilities were evaluated using a 3-(4,5-dimethylthiazol-2-yl)-2,5-phenyltetrazolium bromide (MTT) assay. Briefly, CHO-K1 cells were plated at a density of 5–7 × 10^3^ cells per well in 96-well plates, incubated for 12 h, and treated with various concentrations of BCB (10–500 μg/mL) for 24 h. In other experiments, cells were pretreated with 50–500 μg/mL of BCB for 4 h, 1.5 mM of 4-vinylcyclohexene diepoxide (VCD; Sigma-Aldrich) was added, and cells were incubated for a further 24 h. Viable cells were stained with MTT solution (0.2 mg/mL, Sigma-Aldrich) for 3 h. Formazan crystals were completely dissolved by adding 100 μL dimethyl sulfoxide, and absorbances were measured at 540 nm using a microplate reader (Tecan, Research Triangle Park, NC, USA).

### DPPH radical scavenging activity assay

The ability of BCB to scavenge DPPH (2,2-diphenyl-1-picrylhydrazyl) free radicals was evaluated as previously described [20], and reductions in free radical levels were quantified by measuring absorbance (abs.) at 540 nm. Briefly, various concentrations of BCB (5–500 μg/mL) were mixed with 0.3 mM DPPH ethanol solution and reacted for 30 min in the dark. Absorbances were measured at 515 nm and DPPH free-radical scavenging activities were calculated as follows:

$${\text{Scavenging}}\;{\text{effect}}\left( \% \right) = \left[ {{{\left( {{\text{control}}\;{\text{abs}}.\,{-}\,{\text{sample}}\;{\text{abs}}.} \right)} \mathord{\left/ {\vphantom {{\left( {{\text{control}}\;{\text{abs}}.{-}{\text{sample}}\;{\text{abs}}.} \right)} {{\text{control}}\;{\text{abs}}.}}} \right. \kern-\nulldelimiterspace} {{\text{control}}\;{\text{abs}}.}}} \right] \times 100.$$

### Superoxide anion free-radical scavenging activity assay

The ability of BCB to scavenge superoxide anion free radicals was determined as previously described [20]. Briefly, BCB (5–500 μg/mL) was added to a solution containing 30 mM EDTA (pH 7.4), 30 mM hypoxanthine, and 1.42 mM nitro blue tetrazolium and pre-incubated at room temperature for 3 min. Xanthine oxidase (0.5 U/mL) was then added and allowed to react at room temperature for 20 min. Absorbances were measured at 560 nm.

### Measurement of reactive oxygen species (ROS)

A DCFH-DA (2′,7′-dichlorofluorescein diacetate) assay was used to assess intracellular ROS levels. CHO-K1 cells were plated on a black 96-well plate at 1 × 10^4^ cells/well and incubated with 100 μM hydrogen peroxide (H_2_O_2_) in the presence or absence of BCB (100 μg/mL). After removing medium, 10 μM DCFH-DA was added to each well, and mixtures were incubated at 37 °C for 30 min. Fluorescence intensities were measured at excitation and emission wavelengths of 480 and 530 nm, respectively, using a fluorescence microplate reader (SpectraMAX Gemini, Molecular Devices, Sunnyvale, CA, USA).

### Animal experimental design and treatment

Male and female C57BL/6 mice were purchased from Koatech (Pyeongtaek, Korea). Animals were bred separately by gender and given free access to drinking water and a standard diet and were maintained in a controlled environment under a 12 h light/dark cycle. All experimental procedures were performed according to the guidelines issued by the Animal Research Ethics Committee at Dongguk University Animal Center (IACUC-2016-001).

Experiment 1: Eighteen 4-week-old female mice were randomly divided into three groups: Control group, BCB 100 group, and BCB 300 group. The BCB 100 and BCB 300 groups were orally administered BCB at 100 or 300 mg/kg daily, respectively, for 30 days. At 6 pm local time on day 8 of the 30-day treatment period, all females were exposed to mating with 6-week-old males (ratio 2:1). Days on which vaginal plugs were first discovered, were designated as day 1 of pregnancy. The number of pups born to each mouse was recorded.

Experiment 2: Eighteen 4-week-old female mice were randomly divided into three groups: Control group, RU486 group, and RU486 plus BCB 300 group. Mice in the RU486 plus BCB 300 group were treated with BCB 300 mg/kg for 18–21 days. At 6 pm on day 8 during the 30-day treatment period, all females were exposed to mating with 6-week-old males (ratio 2:1). Days on which vaginal plugs were first discovered, were designated as day 1 of pregnancy. On day 4 of pregnancy, mice in the RU486 and RU486 plus BCB 300 groups were injected subcutaneously with RU486 solution (4 mg/kg, 0.08 mg/100 µL), whereas control group mice were injected with corn oil as vehicle. Seven days after RU486 injection, mice were sacrificed, uterine horns were excised, and numbers of implanted embryos in each uterine horn were counted.

### Western blot analysis

For western blotting, uterus tissue was homogenized in protein lysis buffer consisting of 50 mM Tris-base (pH 7.5), 2 mM EDTA, 1% glycerol, 150 mM NaCl, 10 mM NaF, 10 mM Na-pyrophosphate, 1% NP-40 and protease inhibitors. Cell lysates (30 µg) were loaded and electrophoresed in 10% sodium dodecyl sulfate–polyacrylamide gels and transferred to nitrocellulose membranes. After blocking with 5% skim milk in 0.1% TBS-T for at least 1 h, membranes were incubated with anti-phospho-IκB-α, anti-IκB-α, anti-β-actin (Cell Signaling Technology, Beverly, MA, USA), anti-MT1-MMP, anti-MMP2, anti-COX-2, or anti-iNOS (Abcam, Cambridge, MA, USA) for 16 h at 4 °C. After washing with 0.05% TBS-T, membranes were treated with horseradish peroxidase-conjugated anti-mouse or anti-rabbit IgG for 1 h, and after washing with 0.05% TBS-T, bands were visualized using an enhanced chemiluminescence system (Thermo Fisher Scientific). Band images were obtained using the ChemiDoc™ XRS + system (Bio-Rad, Hercules, CA, USA) and band intensities were analyzed by using Image Lab™ software version 2.0.1 (Bio-Rad).

### Hematoxylin and eosin (H&E) staining

For histological examination, uteri were removed, fixed in 10% formalin overnight, and dehydrated in 70% ethanol. Tissues were then paraffin-embedded, sectioned, and stained with H&E. Three randomly selected sections were chosen for histopathological analysis.

### Statistical analysis

Data were analyzed using the Student’s *t*-test or by one-way ANOVA, and the significances of differences between means were determined using Dunnett’s test or Tukey–Kramer’s multiple comparison test. Null hypotheses of no intergroup differences were rejected for *p*-values of < 0.05. Results are presented as means ± SDs (in vitro) or means ± standard errors (in vivo), and the analysis was performed using SPSS (IBM Corp. Released 2012. IBM SPSS Statistics for Windows, Version 21.0. Armonk, NY: IBM Corp.).

## Results

### Antioxidant and cytoprotective effects of BCB extract

**Fig. 1:**
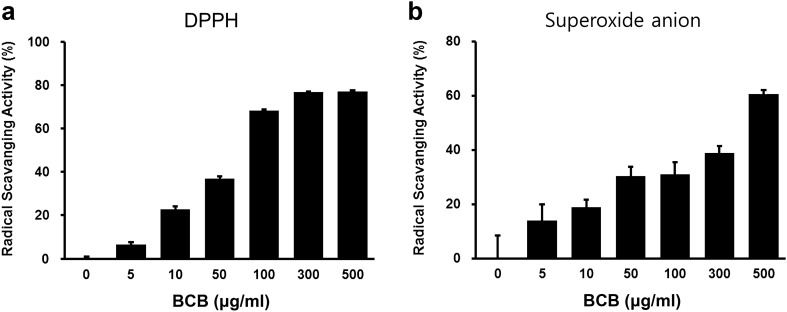
Free radical scavenging activities of BCB extract. **a** DPPH radical scavenging activity of BCB extract. **b** Superoxide anion free-radical scavenging activity of BCB extract

**Fig. 2:**
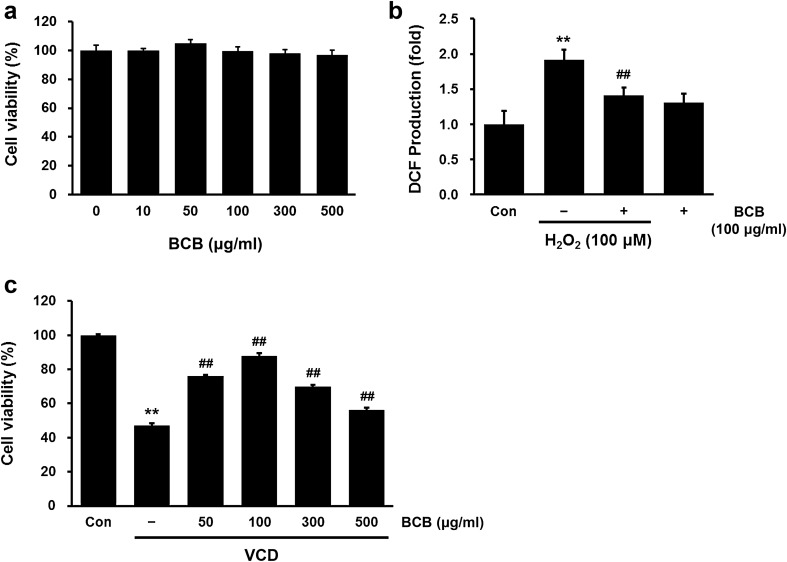
Effects of BCB on ROS generation and its ovotoxicity in CHO-K1 cells. **a** Cytotoxicity of BCB in CHO-K1 cells. Cells were treated with various concentrations of BCB extract (10, 50, 100, 300, or 500 µg/mL) for 24 h. Cell viabilities were measured using an MTT assay. Results are presented as percentages of vehicle-treated controls. **b** Effect of BCB extract on ROS generation. CHO-K1 cells were treated with 100 μM H_2_O_2_ with or without 100 μg/mL BCB. Fold increases in ROS versus vehicle-treated controls, were determined by measuring DCF fluorescence intensities. **c** Effect of BCB extract on ovotoxicity. CHO-K1 cells were pretreated with different concentrations (50–500 μg/mL) of BCB for 2 h and then treated with 1.5 mM VCD for 24 h. (significant vs. vehicle-treated controls, **p < 0.01; significant vs. VCD or H_2_O_2_ treated cells, ^##^*p* < 0.01)

To assess the antioxidant effects of BCB extract, we first evaluated its free-radical scavenging activities. As shown in Fig. 1, BCB extract exhibited strong, dose-dependent, scavenging activities against DPPH and the superoxide anion radical. Next, the cytotoxic effect of BCB on CHO-K1 cells was evaluated using the MTT assay. Results showed that BCB at concentrations up to 500 µg/mL had no toxic effect on CHO-K1 cells (Fig. 2a). To examine the cellular antioxidant capacity of BCB, intracellular ROS levels were measured in H_2_O_2_-treated CHO-K1 cells. H_2_O_2_ treatment significantly increased intracellular ROS levels in CHO-K1 cells as compared with non-treated controls and this increase was suppressed by pretreating cells with 100 µg/mL BCB (Fig. 2b).

In addition, we evaluated whether BCB extract could exert cytoprotective effects using a cell-based screening system that we previously established [21]. VCD (4-vinylcyclohexene diepoxide), an occupational and environmental chemical of interest, can act as an ovotoxicant due to its ability to destroy ovarian follicles selectively [22]. To investigate the protection afforded by BCB extract against VCD, CHO-K1 cells were pretreated with BCB extract at various concentrations (50–500 µg/mL). As shown in Fig. 2c, BCB pretreatment significantly protected CHO-K1 cells against VCD-induced ovotoxicity at all concentrations but with maximal effect at 100 µg/mL.

### BCB administration increased the in vivo implantation rate in mice

**Fig. 3:**
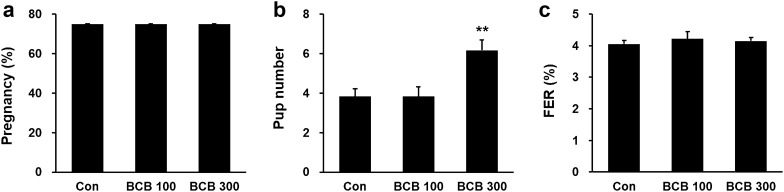
Effects of BCB extract on pregnancy rate and number of newborn pups in normal mice. Female mice were administrated 100 mg/kg or 300 mg/kg of BCB daily for 30 days. After BCB treatment, female mice were mated with normal male mice. Pregnancy rates were measured by counting vaginal plugs (**a**) and numbers of pups (**b**). **c** Food efficiency ratios (FER), expressed as percentages of body weight gains on food intake. Data are presented as means ± SEMs. Groups are: Control (n = 6), BCB 100 mg/kg (BCB 100, n = 6), and BCB 300 mg/kg (BCB 300, n = 6). **p < 0.001 vs. controls

In order to determine the efficacy of BCB treatment on pregnancy rate under normal conditions, female mice were administrated 100 or 300 mg/kg of BCB daily for 30 days, after which, they were mated with normal male mice. Examination showed no significant difference between these two groups in terms of numbers of vaginal plugs (Fig. 3a). However, numbers of pups were significantly higher for 300 mg/kg BCB-treated mice than for 100 mg/kg BCB-treated mice or control mice (Fig. 3b). The food efficiency ratios of mice fed normal or experimental diets were not significantly different (Fig. 3c).

**Fig. 4:**
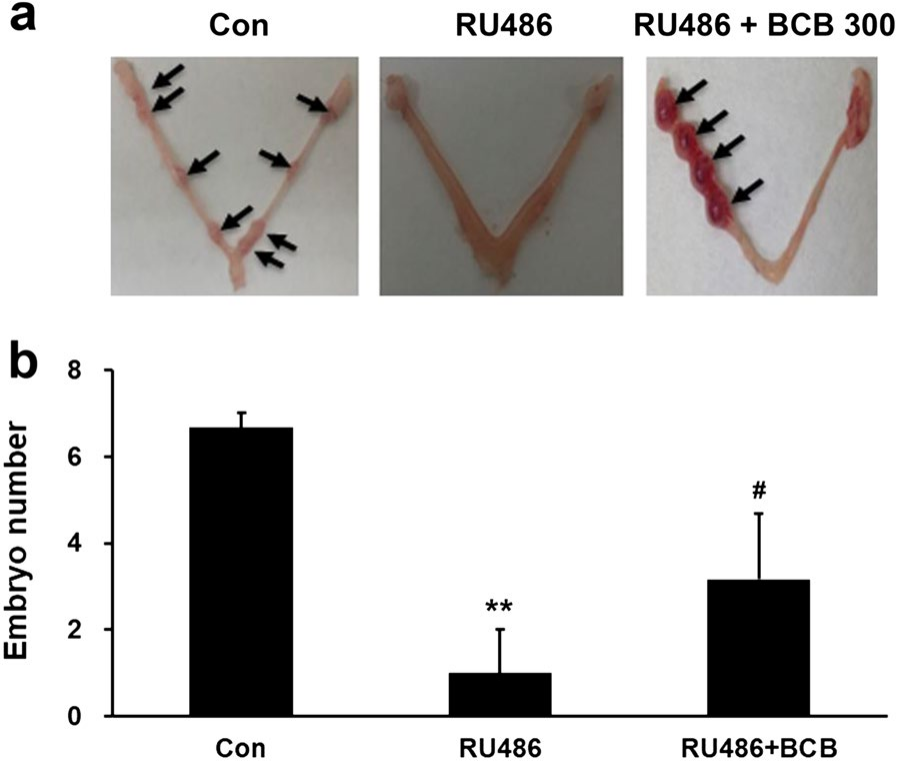
Effects of BCB on in vivo implantation rate in the RU486-induced implantation failure model. Female mice were administrated 300 mg/kg BCB for 18–21 days. On day 8, all females were mated with males, and days when vaginal plugs were first detected were designated as day 1 of pregnancy. The RU486 and RU486 plus BCB 300 groups were injected subcutaneously with RU486 solution (4 mg/kg, 0.08 mg/100 µL), while controls were injected with corn oil as vehicle on day 4 of pregnancy. Seven days after RU486 injection, mice were sacrificed, and uterine horns were excised. **a** Representative photographs of uterine horns showing embryo implantation sites. **b** Quantification of implanted embryos in each uterine horn. Data are presented as means ± SEMs. Groups are: the control group (n = 6), the RU486 group (RU486, n = 6) and the RU486 plus BCB 300 group (RU486 + BCB 300, n = 6). **p < 0.001 vs. controls

Next, we investigated whether the administration of 300 mg/kg BCB daily might promote blastocyst implantation in vivo in our RU486 (4 mg/kg) induced mouse implantation failure model. Numbers of live embryos were recorded on day 8 of pregnancy. The mean number of implanted embryos was markedly lower in the RU486-treated group (0.97 ± 0.98) than in the control group (6.66 ± 0.33) but significantly higher in the RU486 plus BCB group (3.16 ± 1.51) than in the RU486 group (Fig. 4a, b). These results show that BCB improved blastocyst implantation in mice under normal conditions and in our RU486-induced implantation failure model.

### BCB inhibited matrix metalloproteinase (MMP) down-regulation in RU486-treated uteri

**Fig. 5:**
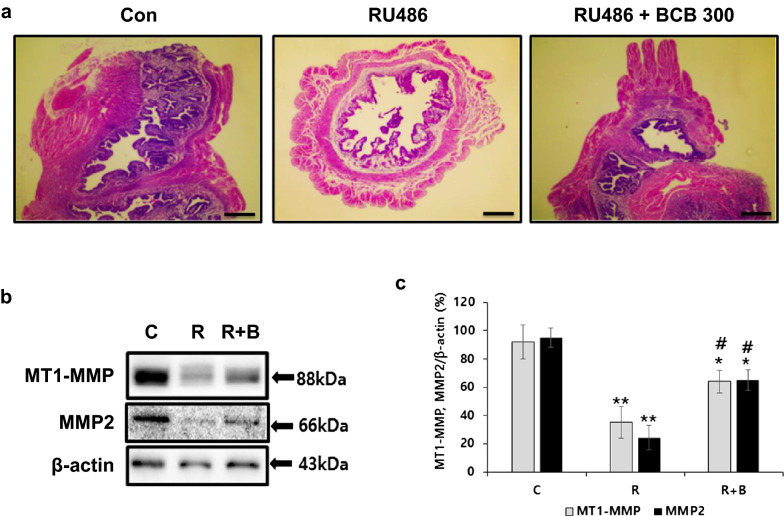
BCB protected against RU486-induced matrix metalloproteinase (MMP) down-regulation in uteri. Female mice were administrated 300 mg/kg BCB for 18–21 days. On day 8, all females were mated with males, and days when vaginal plugs were first detected were designated as day 1 of pregnancy. On day 4 of pregnancy, animals in the RU486 and RU486 plus BCB 300 groups were injected subcutaneously with RU486 solution (4 mg/kg, 0.08 mg/100 µL), while controls were injected with corn oil as vehicle. Seven days after RU486 injection, mice were sacrificed, and uterus tissue was dissected. Endometria were stained with H&E (**a**). Uterus tissues were homogenized and immunoblotted with MT1-MMP and MMP2 antibodies (**b**). Protein levels were normalized versus β-actin (**c**). Data are presented as means ± SEMs. Groups are: the control group (C, n = 3), the RU486 group (R, n = 3), and the RU486 plus BCB 300 group (R + B, n = 3). **p < 0.01, *p < 0.05 vs. controls, ^*##*^*p* < 0.01, ^*#*^*p* < 0.05 vs. the RU486 group

To determine whether BCB affects trophoblast invasion into endometrium, we examined the expression levels of MMP2 and membrane-type 1 MMP (MT1-MMP, also as known MMP14) in mouse uterus tissues. RU486 reduced the expression levels MMP2 and MT1-MMP, and BCB inhibited these down-regulations (Fig. 5b, c). To confirm the effect of BCB treatment on endometrium development, H&E staining of uterus tissue was carried out. Mice in the vehicle-treated control group showed intact endometria, but mice treated with RU486 exhibited damaged/destroyed endometria. However, BCB treatment protected against this RU486-induced destruction of endometrium (Fig. 5a).

### BCB induced COX-2, iNOS, and IκBα expressions in RU486-treated uteri

**Fig. 6:**
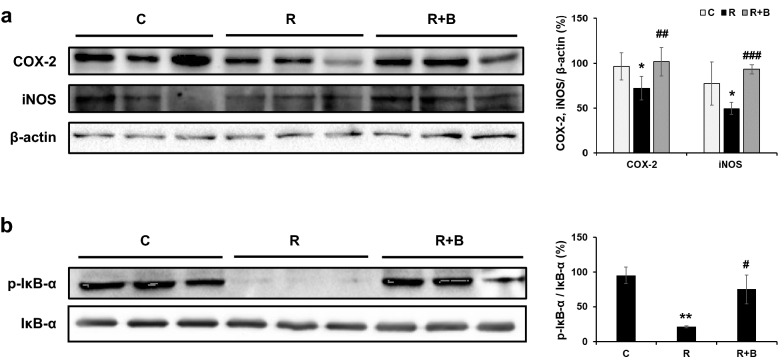
BCB induced the expressions of COX-2, iNOS, and IκBα in RU486-treated mouse uteri. Seven days after RU486 injection, uterus tissues were dissected and immunoblotted with COX-2 and iNOS. Protein levels were normalized versus β-actin (**a**). Uterus tissue lysates were immunoblotted for phosphorylated IκBα and total IκBα and normalized versus total IκBα (**b**). Data are presented as means ± SEMs. Groups are: the control group (**c**, n = 3), the RU486 group (R, n = 3), and the RU486 plus BCB 300 group (R + B, n = 3). **p < 0.01 vs. controls, ^*#*^*p* < 0.05 vs. the RU486 group

In order to investigate the mechanisms responsible for the protective effect of BCB against RU486, we examined the inductions of cyclooxygenase-2 (COX-2) and inducible nitric oxide synthetase (iNOS) in uterus tissues. COX-2 is important for initiating decidualization [23], whereas iNOS is associated with the differentiation process during decidualization [24]. RU486 significantly reduced both COX-2 and iNOS expressions in uterus tissues, but BCB treatment inhibited these reductions (Fig. 6a). Since NF-κB (nuclear factor kappa-light-chain-enhancer of activated B cells) activation is associated with implantation, we examined the expression levels of IκBα in uterus tissues. As shown in Fig. 6b, neither RU486 nor BCB changed the expression of IκBα, but BCB treatment significantly increased IκBα phosphorylation.

### Identification of compounds in BCB extract

**Fig. 7:**
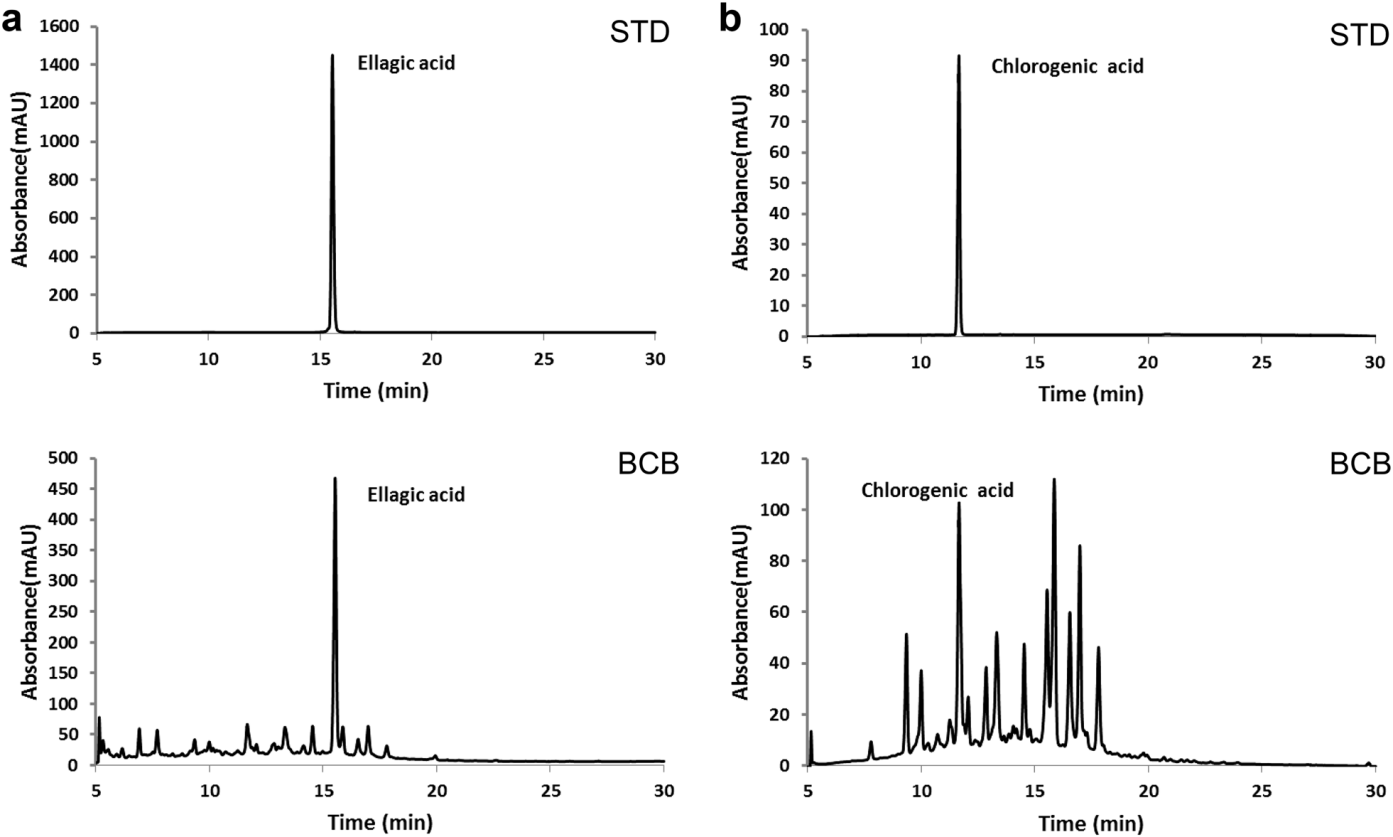
Representative HPLC chromatogram of BCB water extract. Two compounds, ellagic acid (**a**) and chlorogenic acid (**b**) were chosen as marker compounds for quality control purposes. The retention times of ellagic and chlorogenic acids were 15.53 and 11.66 min, respectively

To enable control of BCB extract quality, HPLC analysis was performed using ellagic acid from *Rubi Fructus* and chlorogenic acid from *Lycii Fructus* as controls. The peaks of these two standards appeared at 15.53 min and 11.66 min, respectively (Fig. 7). Concentrations of both acids in BCB extract were determined using standard calibration curves. The amounts of ellagic and chlorogenic acids present in the BCB extract were 3.78 and 0.79 mg/g, respectively.

## Discussion

The present study demonstrates that the administration of a hot water extract of BCB can increase numbers of newborn pups as compared with sham-treated mice under normal conditions and increase the number of implantation sites in pregnant mice treated with RU486. Of the 11 ingredients of BCB, *Cuscutae Semen* and *Rubi Fructus* are often used to treat female infertility as the main herbs used to tonify kidneys in traditional medicine. *Cuscutae Semen* has been shown to increase the number of ovulating ovaries significantly in mice, and *Rubi Fructus* has been reported to improve female gonadal function imbalance and follicle numbers and sizes [25–28]. *Artemisiae Argyi Folium* is also used to treat female infertility and gonadal function imbalance [29]. Moreover, *Ginseng Radix* and *Lycii Fructus* have effects that include the reinforcement of Qi and Yin and the enhancement of embryo implantation and endometrial environment [30, 31]. *Angelicae Gigantis Radix* is an effective treatment for infertility due to its ability to nourish blood [32, 33]. *Dioscoreae Rhizoma* has been shown to prevent abortion, reinforce essences, and tonify kidneys [34], and *Perillae Folium* and *Amomi Fuctus* are representative herbal medicines that can be used to reduce the risk of abortion under threatening conditions [35]. Therefore, the improvement in blastocyst implantation rate observed after the administration of BCB extract may be due to tonifying kidneys, reinforcing Qi, and nourishing the blood. Furthermore, these complementary properties of BCB constituents would appear to be beneficial for embryo implantation. Although we did not elucidate the mechanism responsible for the beneficial effects of BCB on infertility, these findings strongly suggest that BCB has therapeutic potential in the context of poor endometrial receptivity.

NF-κB exists in cytoplasm in the form of inactive NF-κB/IκB complexes formed binding to IκBα [36], and NF-κB activation results from IκBα degradation induced by IκBα phosphorylation and is known to be crucial for mouse embryos development beyond the 2-cell stage [37]. Levels of NF-κB generally increase in the human endometrium premenstrually and during early pregnancy, and these increases may regulate molecules essential for implantation [38, 39]. In the present study, RU486 reduced mean numbers of implanted embryos and IκBα activation, but BCB administration induced the phosphorylation of IκBα in RU486-treated uterus tissues, suggesting that BCB might improve implantation rates by activating the NF-κB/IκBα pathway. However, how BCB regulates IκBα activation in endometrium remains unknown.

NF-κB is critical for the induction of both iNOS and COX-2 in various cells including gingival fibroblasts and endometrial stromal cells [40, 41], and the expression of pro-inflammatory cytokines in uterus throughout the estrous cycle is necessary for embryo receptivity and successful blastocyst implantation [42]. Decidualization describes a series of changes in uterus during the early stages of pregnancy that are necessary for placenta formation and fetal development [43, 44]. In particular, COX-2 is important for the initiation of decidualization and its deletion suppressed fertilization, implantation, and decidualization in a COX-2 deficient mouse model [23]. Nitric oxide (NO) is a key mediator of various physiological functions including vascular functions and inflammatory responses [45], and has been reported to play a crucial role during implantation and pregnancy establishment [46, 47]. In addition, NO may regulate the growth and development of preimplantation embryos [48, 49]. NO is generated by three isoforms of nitric oxide synthase (NOS), and up-regulations of cytokine-inducible NOS (iNOS) and endothelial NOS (eNOS) have been reported in pregnant rodent uteri [50]. In the present study, we also observed that BCB prevented the RU486-induced down-regulations of COX-2 and iNOS in RU486-treated mouse uteri, which suggests BCB-induced IκBα activation might induce COX-2 and iNOS to support implantation and decidualization.

iNOS has also been reported to play an important role in promoting trophoblast invasion and to be particularly abundant at the leading edge of migrating trophoblasts [51], and NO production by iNOS has been shown to regulate trophoblast invasion [51]. Matrix metalloproteinases (MMPs) are indicators of the blastocyst trophoblast invasion of endometrial cells, and MMP expression levels are correlated with trophoblast invasiveness [52]. MMP2 (also as known gelatinase A) is known to degrade ECM and is expressed in endometrium within three days of the onset of blastocyst implantation, which suggests MMP2 might be the primary mediator of endometrium invasion by blastocysts [51]. MT1-MMP, another key MMP, has different functions, which include the degradation of various ECM components and the activation of proMMP-2. Based on this knowledge, we investigated whether BCB affects trophoblast invasion into endometrium. We found that BCB inhibited the down-regulations of MMP2 and MT1-MMP protein levels in RU486-treated uteri and protected against RU486-induced destruction of the endometrium.

ROS, oxidative stress, and antioxidants have been implicated in the establishment and progression of pregnancy including embryo implantation, placental differentiation, and embryo growth [53, 54]. However, ROS can also cause pathological conditions within the female reproductive system [55]. Excess ROS in follicles may overwhelm follicular fluid antioxidant defense, directly damage oocytes, cause defective fertilization, and even when fertilization has been achieved, oxidative stress-induced apoptosis may result in embryo fragmentation, implantation failure, abortion, impaired placentation, or congenital abnormalities [56]. Nicol et al. [57] reported glucose 6-phosphate dehydrogenase prevented oxidative stress-induced embryopathies, and more recently, Qin et al. [58] reported that dehydroepiandrosterone improves endometrium receptivity and enhances embryo implantation by inhibiting the generation of intracellular ROS in endometrial stromal cells. In concert with these findings, in the present study, BCB showed strong scavenging activities against DPPH and superoxide anion radicals and inhibited ROS production in CHO-K1 cells treated with H_2_O_2_. Notably, ellagic acid and chlorogenic acid in BCB have strong antioxidant effects [59, 60]. Although the present study did not demonstrate that the mechanism of action of BCB is directly related to antioxidant activity, evidence suggests that antioxidant activity is a contributory factor.

## Conclusions

We investigated the effects of a hot water extract of BCB on endometrial receptivity in a mouse model of RU486-induced implantation failure. Administration of BCB extract increased numbers of newborn pups as compared with sham-treated mice under normal conditions and increased the number of implantation sites in pregnant mice treated with RU486. In addition, BCB increased the expressions of COX-2 and iNOS via IκBα phosphorylation and up-regulated MMP2 and MT1-MMP levels in uterus implantation sites in RU486-treated mice. Furthermore, BCB exhibited strong anti-oxidative effects in DPPH and superoxide anion free-radicals scavenging assays. These findings show that BCB, a herbal medicine, improved embryo implantation via IκB activation in our murine model and suggest BCB has potential use as a treatment for female infertility.

## Data Availability

The datasets used and/or analysed during the current study are available from the corresponding author on reasonable request.

## Abbreviations

BCB: BaelanChagsangBang
CHO: Chinese hamster ovary
COX-2: Cyclooxygenase-2
DCFH-DA: 2′,7′-Dichlorofluorescein diacetate
DMEM: Dulbecco’s modified Eagle’s medium
DPPH: 2,2-Diphenyl-1-picrylhydrazyl
ECM: Extracellular matrix
EGF: Epidermal growth factor
eNOS: Endothelial nitric oxide synthase
H&E: Hematoxylin and eosin
IκB: Inhibitor of NF-κB
iNOS: Inducible nitric oxide synthetase
MMP: Matrix metalloproteinase
MT1-MMP: Membrane-type 1 MMP
MTT: 3-(4,5-Dimethylthiazol-2-yl)-2,5-phenyltetrazolium bromide
NF-κB: Nuclear factor kappa-light-chain-enhancer of activated B cells
NO: Nitric oxide
NOS: Nitric oxide synthase
ROS: Reactive oxygen species
VCD: Vinylcyclohexene diepoxide

## References

[1] Rashid NA, Lalitkumar S, Lalitkumar PG, Gemzell-Danielsson K (2011). Endometrial receptivity and human embryo implantation. Am J Reprod Immunol.

[2] van Mourik MS, Macklon NS, Heijnen CJ (2009). Embryonic implantation: cytokines, adhesion molecules, and immune cells in establishing an implantation environment. J Leukoc Biol.

[3] Diedrich K, Fauser BC, Devroey P, Griesinger G, Evian Annual Reproduction Workshop G (2007). The role of the endometrium and embryo in human implantation. Hum Reprod Update.

[4] de Mouzon J, Goossens V, Bhattacharya S, Castilla JA, Ferraretti AP, Korsak V, Kupka M, Nygren KG, Nyboe Andersen A, European Ivf-monitoring Consortium ftESoHR (2010). Assisted reproductive technology in Europe, 2006: results generated from European registers by ESHRE. Hum Reprod.

[5] Kudesia R, Chernyak E, McAvey B (2017). Low fertility awareness in United States reproductive-aged women and medical trainees: creation and validation of the Fertility & Infertility Treatment Knowledge Score (FIT-KS). Fertil Steril.

[6] Kim SH, Jo J, Kim DI (2017). The effectiveness, safety, and economic evaluation of Korean medicine for unexplained infertile women: a multi-center, prospective, observational study protocol. Medicine.

[7] Fang C, Huang R, Li TT, Jia L, Li LL, Liang XY (2013). Day-2 and day-3 sequential transfer improves pregnancy rate in patients with repeated IVF-embryo transfer failure: a retrospective case-control study. Reprod Biomed Online.

[8] Gao W, Tang X, Chen Z, Guo Y, Wang L, Zhang M, Huang G (2013). Effects of acupuncture on CCL2 and CXCL8 expression and the subset of uNK cells in rats with embryo implantation failure. Evid Based Complement Alternat Med.

[9] Jeong JC, Choi MS, Yoon SH, Kim DI (2015). Analysis of the result of Korean medicine treatment for female subfertility using herbal medicine, acupuncture and moxibustion treatment. J Korean Med.

[10] Chung TW, Park MJ, Lee H, Kim KJ, Kim CH, Choi HJ, Ha KT (2019). Enhancement of endometrial receptivity by cnidium officinale through expressing LIF and integrins. Evid Based Complement Alternat Med.

[11] Choi HJ, Chung TW, Park MJ, Lee KS, Yoon Y, Kim HS, Lee JH, Kwon SM, Lee SO, Kim KJ (2016). *Paeonia lactiflora* enhances the adhesion of trophoblast to the endometrium via induction of leukemia inhibitory factor expression. PLoS ONE.

[12] Burks-Wicks C, Cohen M, Fallbacher J, Taylor RN, Wieser F (2005). A Western primer of Chinese herbal therapy in endometriosis and infertility. Womens Health.

[13] Zhou J, Qu F (2009). Treating gynaecological disorders with traditional Chinese medicine: a review. Afr J Tradit Complement Altern Med.

[14] Yang DH, Kang JH, Park YB, Park YJ, Oh HS, Kim SB (2013). Association rule mining and network analysis in oriental medicine. PLoS ONE.

[15] Kim DI, Choi MS, Pak SC, Lee SB, Jeon S (2012). The effects of Sutaehwan-Gami on menopausal symptoms induced by ovariectomy in rats. BMC Complement Altern Med.

[16] Fiala C, Gemzel-Danielsson K (2006). Review of medical abortion using mifepristone in combination with a prostaglandin analogue. Contraception.

[17] Sarkar NN (2005). The potential of mifepristone (RU-486) as an emergency contraceptive drug. Acta Obstet Gynecol Scand.

[18] Huang DM, Huang GY, Lu FE (2010). Effects of Bushenyiqihexue formula on the endometrial gland apoptosis in mice with blastocyst implantation dysfunction. J Tradit Chin Med.

[19] Osuka S, Nakanishi N, Murase T, Nakamura T, Goto M, Iwase A, Kikkawa F (2019). Animal models of polycystic ovary syndrome: a review of hormone-induced rodent models focused on hypothalamus-pituitary-ovary axis and neuropeptides. Reprod Med Biol.

[20] Park JY, Kwon YW, Lee SC, Park SD, Lee JH (2017). Herbal formula SC-E1 suppresses lipopolysaccharide-stimulated inflammatory responses through activation of Nrf2/HO-1 signaling pathway in RAW 264.7 macrophages. BMC Complement Altern Med.

[21] Nam EY, Kim SA, Kim H, Kim SH, Han JH, Lee JH, Kim DI (2016). Akt activation by Evodiae Fructus extract protects ovary against 4-vinylcyclohexene diepoxide-induced ovotoxicity. J Ethnopharmacol.

[22] Kappeler CJ, Hoyer PB (2012). 4-Vinylcyclohexene diepoxide: a model chemical for ovotoxicity. Syst Biol Reprod Med.

[23] Lim H, Paria BC, Das SK, Dinchuk JE, Langenbach R, Trzaskos JM, Dey SK (1997). Multiple female reproductive failures in cyclooxygenase 2-deficient mice. Cell.

[24] Thienel T, Chwalisz K, Winterhager E (2002). Expression of MAPkinases (Erk1/2) during decidualization in the rat: regulation by progesterone and nitric oxide. Mol Hum Reprod.

[25] Cho SK, Kim YS, Jang JB, Lee KS (2002). The effects of administration of semen cuscutae on ovulation and developmental competence in mice. Korean J Orient Med.

[26] Shin MK (2000). Clinical traditional herbalogy.

[27] Kim HC, Lee SI (1991). The comparison research on effect of Rubi Fructus. Korean J Herbol.

[28] Kim BS, Park YK, Kang BS (2001). The effect of Rubi Fructus on the ovulation and ovary in rats. Korean J Herbol.

[29] Ahn IS, Kim DI, Choi MS, Jang SW, Jeong JC (2013). A study on factors influencing pregnancy in the pilot project for Korean medical treatment for subfertility. J Korean Obstet Gynecol.

[30] Bae WJ, Lee JM, Lee CH, Cho JH, Jang JB, Lee KS (2008). Effects of *Allii tuberosi* semen, *Ginseng radix *alba and *Morindae officinalis* radix extract on reproductive capacities in mice. J Orient Obstet Gynecol.

[31] Ahn KH, Lee SJ, Choi CM, Yoo SK (2005). Effects of Guisinhwan on the ovulation and ovary in rats. J Orient Obstet Gynecol.

[32] Ryu KJ, Cho SH (2011). Effects of *Angelicae gigantis* radix on gene expression of ovarian tissue in polycystic ovary syndrome rats. J Orient Obstet Gynecol.

[33] Kim HW, Choi E, Chung H, Joung YM, Shin DS, Cho SI (2011). Effects of *Angelicae gigantis* radix (AGR) on polycystic ovary induced by estradiol valerate in rats. Korean J Herbol.

[34] Kim SR, Jeong JH, You DY (1999). A study on the effect of an Jeonyi Cheon Tang on sex hormones changes and lipid metabolism in the ovariectomized rats. J Korean Obstet Gynecol.

[35] Kim CS, Park HM, Lee SD, Lee JW, Kim PG, Shin HT (2007). The effects of the administration on oriental medicine, Antaeeum, in the pregnant rat and their fetuses. Korean J Environ Health.

[36] Schuster M, Annemann M, Plaza-Sirvent C, Schmitz I (2013). Atypical IkappaB proteins—nuclear modulators of NF-kappaB signaling. Cell Commun Signal.

[37] Nishikimi A, Mukai J, Yamada M (1999). Nuclear translocation of nuclear factor kappa B in early 1-cell mouse embryos. Biol Reprod.

[38] King AE, Critchley HO, Kelly RW (2001). The NF-kappaB pathway in human endometrium and first trimester decidua. Mol Hum Reprod.

[39] Sakowicz A (2018). The role of NFkappaB in the three stages of pregnancy—implantation, maintenance, and labour: a review article. BJOG.

[40] Nakao S, Ogtata Y, Shimizu E, Yamazaki M, Furuyama S, Sugiya H (2002). Tumor necrosis factor alpha (TNF-alpha)-induced prostaglandin E2 release is mediated by the activation of cyclooxygenase-2 (COX-2) transcription via NFkappaB in human gingival fibroblasts. Mol Cell Biochem.

[41] Shukla V, Kaushal JB, Sankhwar P, Manohar M, Dwivedi A (2019). Inhibition of TPPP3 attenuates beta-catenin/NF-kappaB/COX-2 signaling in endometrial stromal cells and impairs decidualization. J Endocrinol.

[42] Dekel N, Gnainsky Y, Granot I, Racicot K, Mor G (2014). The role of inflammation for a successful implantation. Am J Reprod Immunol.

[43] Aikawa S, Kano K, Inoue A, Wang J, Saigusa D, Nagamatsu T, Hirota Y, Fujii T, Tsuchiya S, Taketomi Y (2017). Autotaxin-lysophosphatidic acid-LPA3 signaling at the embryo-epithelial boundary controls decidualization pathways. EMBO J.

[44] Lim HJ, Wang H (2010). Uterine disorders and pregnancy complications: insights from mouse models. J Clin Invest.

[45] Ignarro LJ (1999). Nitric oxide: a unique endogenous signaling molecule in vascular biology. Biosci Rep.

[46] Natuzzi ES, Ursell PC, Harrison M, Buscher C, Riemer RK (1993). Nitric oxide synthase activity in the pregnant uterus decreases at parturition. Biochem Biophys Res Commun.

[47] Chwalisz K, Garfield RE. Implantation rates after in vitro fertilization, treatment of infertility and early pregnancy loss with a nitric oxide donor alone or in combination with progesterone, and a method for contraception with nitric oxide inhibitors. US Patent Number 6,040,340. US; 2000.

[48] Gouge RC, Marshburn P, Gordon BE, Nunley W, Huet-Hudson YM (1998). Nitric oxide as a regulator of embryonic development. Biol Reprod.

[49] Chen HW, Jiang WS, Tzeng CR (2001). Nitric oxide as a regulator in preimplantation embryo development and apoptosis. Fertil Steril.

[50] Purcell TL, Given R, Chwalisz K, Garfield RE (1999). Nitric oxide synthase distribution during implantation in the mouse. Mol Hum Reprod.

[51] Harris LK, McCormick J, Cartwright JE, Whitley GS, Dash PR (2008). S-nitrosylation of proteins at the leading edge of migrating trophoblasts by inducible nitric oxide synthase promotes trophoblast invasion. Exp Cell Res.

[52] Staun-Ram E, Goldman S, Gabarin D, Shalev E (2004). Expression and importance of matrix metalloproteinase 2 and 9 (MMP-2 and -9) in human trophoblast invasion. Reprod Biol Endocrinol.

[53] Al-Gubory KH, Fowler PA, Garrel C (2010). The roles of cellular reactive oxygen species, oxidative stress and antioxidants in pregnancy outcomes. Int J Biochem Cell Biol.

[54] Ruder EH, Hartman TJ, Reindollar RH, Goldman MB (2014). Female dietary antioxidant intake and time to pregnancy among couples treated for unexplained infertility. Fertil Steril.

[55] Agarwal A, Gupta S, Sharma R (2005). Oxidative stress and its implications in female infertility—a clinician's perspective. Reprod Biomed Online.

[56] Sharma RK, Agarwal A (2004). Role of reactive oxygen species in gynecologic diseases. Reprod Med Biol.

[57] Nicol CJ, Zielenski J, Tsui LC, Wells PG (2000). An embryoprotective role for glucose-6-phosphate dehydrogenase in developmental oxidative stress and chemical teratogenesis. FASEB J.

[58] Qin A, Qin J, Jin Y, Xie W, Fan L, Jiang L, Mo F (2016). DHEA improves the antioxidant capacity of endometrial stromal cells and improves endometrium receptivity via androgen receptor. Eur J Obstet Gynecol Reprod Biol.

[59] Tosovic J, Markovic S (2019). Antioxidative activity of chlorogenic acid relative to trolox in aqueous solution—DFT study. Food Chem.

[60] Tosovic J, Bren U (2020). Antioxidative action of ellagic acid-A kinetic DFT study. Antioxidants.

